# Social and Emotional Fears and Worries Influencing the Quality of Life of Female Celiac Disease Patients Following a Gluten-Free Diet

**DOI:** 10.3390/nu10101414

**Published:** 2018-10-03

**Authors:** Wioleta Zysk, Dominika Głąbska, Dominika Guzek

**Affiliations:** 1 Department of Organization and Consumption Economics, Faculty of Human Nutrition and Consumer Sciences, Warsaw University of Life Sciences (SGGW-WULS), 159C Nowoursynowska Street, 02-787 Warsaw, Poland; wioleta_zysk@sggw.pl; 2 Department of Dietetics, Faculty of Human Nutrition and Consumer Sciences, Warsaw University of Life Sciences (SGGW-WULS), 159C Nowoursynowska Street, 02-787 Warsaw, Poland; dominika_glabska@sggw.pl

**Keywords:** celiac disease (CD), quality of life (QoL), gluten-free diet, disease duration, place of residence, educational level, economic status, body mass index (BMI)

## Abstract

The gluten-free diet is effective in the majority of celiac disease (CD) patients, but it is burdensome and may influence quality of life (QoL). The aim of the study was to analyze the social and emotional fears and worries influencing the QoL of female CD patients following a gluten-free (GF) diet, as well as to indicate the sociodemographic interfering factors. The study was conducted on a group of 251 female CD patients, while emotional, social and worries subscales of the Celiac Disease Questionnaire (CDQ) were applied, as well as purchase-related emotions and behaviors were assessed. Respondents declaring worse economic status obtained significantly lower scores in the emotional, social and worries subscales of the CDQ than respondents declaring better economic status, while for other factors (CD duration, GFD adherence, BMI, place of residence and educational level) no significant association was stated in the multi-factor analysis. Moreover, respondents declaring worse economic status more often declared that a bad mood affected their purchase decisions than did respondents declaring better economic status. It was stated, that the economic status of CD patient could be one of the most important factors influencing their social and emotional fears and worries. It may be supposed that low economic status may lead some CD patients to choose to relieve stress by purchasing GF products instead of other products.

## 1. Introduction

Celiac disease (CD) is diagnosed in almost 1% of the global population, but a high percentage of affected individuals are undiagnosed [1]. It is an autoimmune disease, defined as an inflammatory disorder of the small intestine characterized by persistent gluten protein intolerance [2,3]. In CD patients, gluten exposure leads to enteropathy with mucosal surface damage and, as a consequence, nutrient malabsorption [4]. The only therapeutic approach for CD is a gluten-free diet (GFD) [5], and strict adherence is necessary to normalize the mucous membrane of the small intestine and to improve the impaired absorption [6].

GFD is effective in the majority of CD patients, but it is burdensome and may influence the quality of life (QoL) of patients [6]. After diagnosis, some patients may feel overwhelmed and the disease itself may affect their QoL [7]. Also, following GFD is commonly associated with certain difficulties in coping with information overload and in the implementation of recommendations influencing dietary behaviors [5]. As a result, the two main aspects of the QoL of CD patients, both associated with specific eating habits, concern the quality of their social and family life.

One of the most common difficulties for CD patients is the eating-out experience. CD patients are concerned about the possibility of finding gluten-free (GF) food products or dishes in restaurants, as well as about the possibility of cross-contamination of available GF products [5]. Moreover, they may feel socially isolated when they must decline an invitation to a regular restaurant [8]. In the study by Zarkadas et al. [9], almost 80% of members of the Canadian Celiac Association stated avoiding restaurants due to this reason, while in a further study by Zarkadas et al. [10] over 70% declared being frustrated as a result of limited choices on restaurant menus, as well as almost 90% declared limited choices at fast food restaurants and almost 80%—a limited choice of restaurants. However, knowledge about GFD, as well as the GF product market has recently been increasing sharply [11]. Therefore, the situation for CD patients may be changing in the context of available restaurant GF products and dishes.

It is crucial to involve family members to prevent interference with their relationships with the CD patient, because GFD inevitably affects eating practices at home. A patient on a GFD needs assurance that gluten is absent from a product or dish, as well as that GF products are not contaminated [12]. Moreover, adherence to GFD is difficult for some patients; and therefore, the support of their relatives is also important [13].

This situation may also be gender-related, as in many countries women are responsible for their family meal preparation, and also for the family grocery shopping decisions [14]. This is observed inter alia in Poland [15] and has been indicated in Polish studies as an important determinant for consumer behaviors [16]. Given that for CD men family support in following the diet is natural, CD women may suffer from a lack of such support and thus their need for support may be higher than it is for men [17]. Furthermore, some CD patients may also experience psychological, emotional and economic stress caused by everyday implications of GFD [8]. In some studies, this percentage is higher in women than in men [17].

Many studies analyzed the gastrointestinal symptoms of CD, CD screening and management strategies, and GFD adherence in CD patients. However, only limited data describing the QoL associated with the social and emotional components of GFD are available for CD patients [18]. Therefore, the aim of the study was to analyze the social and emotional fears and worries influencing the QoL of female CD patients following a GFD, as well as to indicate the sociodemographic interfering factors.

## 2. Materials and Methods

### 2.1. Ethics Approval Statement

The study was conducted according to the guidelines laid down in the Declaration of Helsinki. The study was approved by the Ethics Committee of the Faculty of Human Nutrition and Consumer Sciences of the Warsaw University of Life Sciences (No. 20/2017; 19.06.2017). All the participants provided their informed consent to participate.

### 2.2. Participants

The study was conducted on a group of CD patients. Participants were recruited online by a nationwide search via local CD and GFD societies. The combined methods of purposive sampling (patients with CD diagnosed and confirmed) and network sampling (local CD and GFD societies) were applied.

Inclusion criteria were:

- women;
- aged ≥ 18 years old;
- CD diagnosed and biopsy-confirmed by a physician;
- declared regular meal preparation at home;
- declared regular purchase of GF products.

Exclusion criteria were:

- any data missing in the completed QoL questionnaires;
- lack of informed consent to participate.

**Figure 1:**
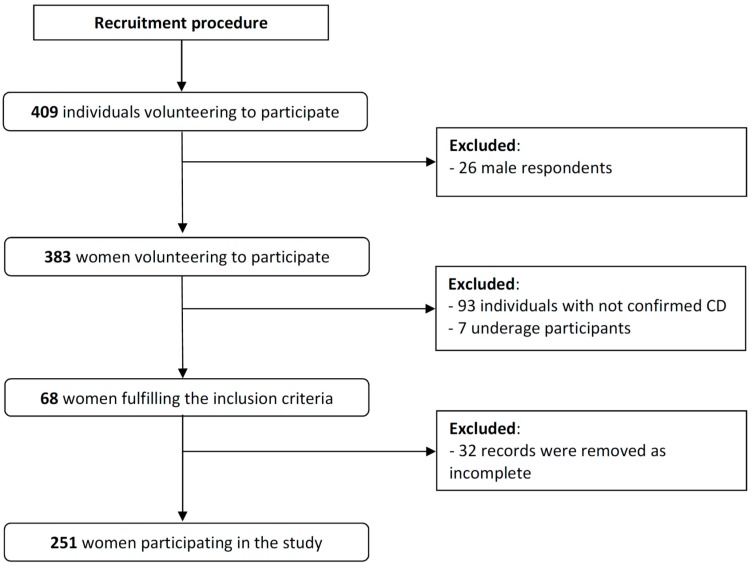
Participants inclusion to the study.

As a result, 158 of the 409 volunteers were excluded due to not meeting the inclusion criteria (Figure 1).

### 2.3. Study Design

Subjects were interviewed using the Computer-Assisted Web Interview (CAWI) method. The questionnaire applied was divided into two main parts. The first part included the Celiac Disease Questionnaire (CDQ), developed by Häuser et al. [19] on the basis of the Health-Related Quality of Life Questionnaire (HRQOL), as a specific form for adult CD patients. The second part included questions regarding purchase-related emotions and behaviors, as well as attitudes toward GF product attributes.

CDQ consists of four subscales–emotional subscale, social subscale, worries subscale and gastrointestinal subscale [19]. The Polish version of CDQ was applied in the present study, while it was, in some points, modified due to the aim and scope of the study. In the study, emotional, social and worries subscales were included, as the aim of the study was to assess the social and emotional fears and worries influencing the QoL, and not the gastrointestinal symptoms. Moreover, from the worries subscale, two questions directly associated with medical conditions were excluded on the basis of the pilot study so as not to cause discomfort and anxiety in the respondents and because they were not associated with the aim of the study. The excluded questions were related to cancer as a CD complication (“How many times during the last 2 weeks did you worry about or were you afraid of getting cancer as a result of your CD?”) and to the possibility of CD inheritance (“How many times during the last 2 weeks were you concerned that your children could inherit or may have inherited your CD?”). A similar approach was applied in the study by Tovoli et al. [20], as a cancer-related question was also excluded. For test-retest reproducibility of the applied tool, a priori assumed criteria of Pearson correlation coefficients (R > 0.7, as recommended by DeVellis [21]) were obtained. The present study represents the first time the tool has been applied on a Polish population. This was based on the consent of the author of the CDQ [19] to apply the tool, obtained via e-mail.

Evaluation of the quality of data obtained on the basis of the CDQ included the floor and ceiling effects, item internal consistency and internal consistency reliability assessment (Cronbach’s alpha coefficient > 0.7, as recommended by DeVellis [21]).

Among the questions regarding purchase-related emotions and behaviors, as well as attitude toward GF product attributes, there were 10 close-ended questions: Two of these were associated with purchase-related emotions and behaviors (included in the present study) and eight were associated with attitude toward GF product attributes (not related to the aim of the present study). The questions were formulated as follows: (1) “In the past 2 weeks, how many times did your mood affect your purchase decisions associated with GF products?”; (2) “In the past 2 weeks, how many times did you buy GF products to improve your mood?”. For these questions, GF products were defined as products naturally free of gluten, pre-packed GF products and non-pre-packed GF products, such as those served in restaurants, according to Regulation (EU) No 828/2014 [22].

The additional part of the questionnaire included questions related to disease characteristics, including CD duration since diagnosis and GFD adherence. CD duration since diagnosis was declared by respondents in months/years. GFD adherence was assessed on a 4-point scale, as had been applied for GFD adherence in CD patients by inter alia Häuser et al. [2], van Hess et al. [23], or Kautto et al. [24]. In order to facilitate assessment, the four categories were translated into the following descriptions of adherence to GFD: (1) very poor; (2) good, but occasionally eat dishes containing gluten (at home or outside home); (3) very good, but occasionally eat dishes containing gluten (only outside home); (4) excellent. The translation of categories into descriptions was performed on the basis of commonly stated difficulties in adhering to GFD outside the home [13] and its influence on general GFD adherence [25]. An additional category was added for respondents who refused or were not able to assess their behavior.

The other questions in the additional part of the questionnaire were related to height and weight, in order to calculate Body Mass Index (BMI) [26]. Moreover, questions about the sociodemographic characteristics of respondents were included: gender, age, educational level, place of residence, occupational status and individual economic status (self-assessment).

### 2.4. Statistical Analysis

The statistical analysis included assessment of the normality of distribution (conducted using the Shapiro-Wilk test). Differences between groups were identified using the multi-factor analysis of variance (ANOVA) for the main effects and interaction effects. The Cronbach’s alpha coefficient was applied to verify the internal reliability of data. The level of *p* ≤ 0.05 was accepted as significant. The analysis was conducted using Statistica software version 8.0 (StatSoft Inc., Tulsa, OK, USA).

## 3. Results

### 3.1. Characteristics of the Analyzed Group of Patients

The characteristics of the CD patients are presented in Table 1. The median age of the patients was 33 years old (range 18–63; non-parametric distribution of data). Over half of the respondents were highly educated and they reported that they were employed (77%) or they were students (10%) while the study was being conducted. Furthermore, a high level of self-reported adherence to GFD was observed. Slightly over half of the respondents lived in big cities (cities over 100,000 inhabitants–size corresponding to the size of a Polish provincial capital).

### 3.2. Descriptive Statistics for the CDQ

The emotional, social and worries subscales data in the analyzed group of CD individuals are presented in Table 2. The floor effect was found to be negligible for all subscales, whereas the ceiling effect for the social subscale was higher, which suggests a tendency to score near the top of the subscale. The Cronbach’s alpha coefficient demonstrated the good internal reliability of the data (≥0.7). In the case of item-scale correlation, the results were attributed to consistency levels from acceptable to good, and this was considered satisfactory [27].

### 3.3. Social and Emotional Fears and Worries Influencing the QoL of Female CD Patients Following a GFD

The scores for emotional subscale of the CDQ categorized by CD duration, GFD adherence, BMI and sociodemographic characteristics are presented in Table 3. Respondents declaring worse economic status obtained significantly lower scores in the subscale than respondents declaring better economic status, while for other factors no significant association was stated in the multi-factor analysis.

The scores for social subscale of the CDQ categorized by CD duration, GFD adherence, BMI and sociodemographic characteristics are presented in Table 4. Respondents declaring worse economic status obtained significantly lower scores in the subscale than respondents declaring better economic status, while for other factors no significant association was stated in the multi-factor analysis.

The scores for worries subscale of the CDQ categorized by CD duration, GFD adherence, BMI and sociodemographic characteristics are presented in Table 5. Respondents declaring worse economic status obtained significantly lower scores in the subscale than respondents declaring better economic status, while for other factors no significant association was stated in the multi-factor analysis.

The purchase-related emotions and behaviors data regarding question about mood affecting purchase decisions categorized by CD duration, GFD adherence, BMI and sociodemographic characteristics are presented in Table 6. Respondents declaring worse economic status more often declared that their mood affected their purchase decisions (obtained significantly lower scores for the Likert scale) than did respondents declaring better economic status, while for other factors no significant association was stated in the multi-factor analysis.

The purchase-related emotions and behaviors data regarding question about purchasing GF products because of mood categorized by CD duration, GFD adherence, BMI and sociodemographic characteristics are presented in Table 7. For the analyzed factors, no significant association was stated in the multi-factor analysis.

## 4. Discussion

### 4.1. The Influence of CD Duration and Gender on the QoL of Patients

CD patients experience, due to their diagnosis and applied therapy, a number of problems related to their physical activity, lifestyle and eating behaviors that may affect their general QoL. However, in the review by Kurppa et al. [29], it was stated that additional factors that may affect health-related QoL of CD patients are: age at diagnosis, gender, comorbidities, dietary compliance, availability of GF products, and general knowledge about CD.

Nevertheless, the influence of some factors, e.g., age at diagnosis, on the QoL of CD patients may be contradictory, in various studies. Häuser et al. [2] and Zarkadas et al. [9] reported that diagnosis of CD at a younger age may lead to better health-related QoL, whereas Ciacci et al. [30] and Wagner et al. [31] indicated the reverse relationship.

In our own study, in order to verify the influence of CD duration, a similar assessment was conducted. Those patients with a CD duration below and over 3 years were compared, as 3 years of following a GFD is indicated as the time needed for CD adults to achieve mucosal recovery [32] and, moreover, the first 3 years of disease duration were defined in a cohort study of CD patients as a period characterized by increased mortality [33]. However, neither for emotional, social or worries subscales nor for purchase-related emotions and behaviors was the influence of disease duration reported.

Therefore, in the studied group, other factors, including sociodemographic ones, had to be considered. One of the important sociodemographic factors is gender, for which, in general, CD women experience poorer general well-being than CD men [34]. A similar situation has also been observed for other diseases or health conditions, such as hypertensive patients [35], patients undergoing coronary angiography [36], or patients with diagnosed bipolar disorder [37]. In all these studies, the QoL was measured using HRQOL questionnaires and was lower for female than for male respondents.

### 4.2. The Influence of GFD Adherence and BMI on the QoL of Patients

Considering the problem of the low QoL of female patients, the aim of the study was to assess the social and emotional fears and worries influencing the QoL of CD patients, in particular for the sub-group of female respondents, being those who experience especially decreased QoL, in comparison with male respondents. Moreover, as following a GFD is not only the recommended approach [38] but also influences the general QoL of CD individuals, a homogenous group of CD female patients following the GFD was studied. 

In general, the majority of CD patients have a good QoL while they follow the GFD [29], but untreated patients have a significantly poorer QoL [39]. However, while general diet following influences the QoL of CD patients, dietary adherence level does not have such influence, as comparisons of non-adherent and strictly adherent patients have not demonstrated any significant differences [40]; neither were such differences confirmed in our analyzed group. This may be explained by the fact that even adherent CD patients may have some gastrointestinal symptoms, as may non-adherent ones [41]; so in general, they may be used for such symptoms.

In general, there have so far been no studies analyzing the influence of BMI on the QoL of CD patients. However, for other diseases, such an influence is sometimes stated, and in the context of CD, results observed for gastrointestinal diseases may be used for comparative purposes. For inflammatory bowel diseases, a longitudinal natural history data study showed that obesity was associated with decreasing QoL [42]. Similarly, in a study of an elderly patients group diagnosed with inflammatory bowel diseases, both being underweight and obese were associated with unfavorable health-outcomes, since being underweight was associated with a lower QoL and obesity was associated with a higher risk of depression [43]. The negative impact of both low and high body mass may be confirmed by results from a cross-sectional analysis of the Swiss Inflammatory Bowel Diseases cohort study, as QoL was globally affected in anorexic patients, while in obese ones decreases in the systemic QoL and social QoL scores were stated [44]. These observations for inflammatory bowel diseases are in agreement with the results observed for the general population, as the meta-analysis of Ul-Haq et al. [45] provided evidence that QoL is decreased in obese individuals.

However, not all studies conducted for gastrointestinal diseases indicate such associations. For women with irritable bowel syndrome, the reverse relationship was observed and normal body mass was associated with lower QoL when compared with overweight respondents [46]. Similarly, our study did not indicate any influence of BMI on the emotional, social and worries subscales, or on purchase-related emotions and behaviors in CD patients. 

### 4.3. The Influence of Sociodemographic Characteristics on the QoL of Patients 

The number of patients with diagnosed CD is constantly increasing [47] and with this the necessity for permanent changes to eating behaviors which may impacts upon lifestyles. For our group of female CD patients following the GFD, the main sociodemographic factor influencing their social and emotional fears and worries was economic status. Neither for place of residence, nor for educational level the influence was stated, while economic status influenced not only the emotional, social and worries subscales of the CDQ, but also respondents declaring worse economic status more often stated that a mood affected their purchase decisions.

In the literature, there is almost no information about the influence of socioeconomic status on the factors associated with the QoL of CD patients. This aspect is especially important, given that the prices of GF products are higher than the prices of regular ones. However, a number of people follow a strict GF diet, even if they have no specific medical conditions that require this, so the market for GF products is currently increasing [48]. Such a situation, in which the market is increasing (generating higher supply and higher accessibility) but where the prices are still high is very specific and may be burdensome for low-income CD individuals. This is confirmed by other authors, who have reported that GFD may be expensive and challenging for CD patients [49].

The results of our study suggest that not only do low economic status patients not reduce seeking stress-relief in shopping, a strategy commonly observed in the general population [50], but rather they may purchase GF products even more often to improve their mood than do other respondents. This may result from the fact that a properly followed GFD is the primary therapeutic approach [38], so CD patients consciously choose to spend extra money on GF products, instead of spending on other goods. Thus, they consciously purchase the GF products that they need, but at the same time, they sub-consciously satisfy their other needs.

At the same time, lower scores for the emotional, social and worries subscales indicate the lower QoL of CD patients of lower economic status than for those of higher economic status—a situation that is not surprising. However, lower QoL in low-income CD patients may induce the above stress-relief response, while their economic status means that they mainly go grocery shopping, especially for GF products.

The lack of influence of CD duration, GFD adherence and BMI, as well as place of residence and educational level may be associated with the prominent influence of other interfering factors in the case of CD, such as the influence of economic status. This means that economic status in CD patients must be treated as a dominant factor, creating observable associations and influencing social and emotional fears and worries.

### 4.4. Limitations of the Study and Future Perspectives for the Research

Although the observed associations are interesting, the potential limitations of our study should be noted. Other factors that were not analyzed in the present study may also influence the QoL of CD patients. Among such factors, age and age at diagnosis may be highlighted, as in pediatric patients it has been observed that age is associated with GFD adherence [51,52], as is age combined with educational level at diagnosis [53]. Another factor that may influence the QoL of CD patients may be dietary counseling, as GFD adherence may result from the patient’s knowledge and understanding of the rules of GFD [54].

A self-report method carries with it several limitations associated with three areas—respondents, instrument of data collection and contextual factors. Moreover, our relatively small size sample does not allow generalization of our findings.

It would be interesting to conduct a similar study with a group of male CD patients, in order to compare results for men and women, as it has been claimed that there are general gender-related differences in the clinical presentation of CD [55]. Also, conducting a similar study with a group of children would be interesting, as it is especially challenging for children, in particular teenagers, to adhere to GFD [56].

## 5. Conclusions

1. The economic status of a CD patient could be one of the most important factors influencing their social and emotional fears and worries.
2. Although the low economic status of CD patients may lead to a lower QoL in terms of social and emotional fears and worries, it does not reduce the purchasing of GF products to improve mood.
3. It may be supposed that low economic status may lead some CD patients to choose to relieve stress by purchasing GF products instead of other products.

## Figures and Tables

**Table 1:** Characteristics of the study sample (*n* = 251).

Characteristics	Category	Number of Respondents (%)
CD duration	Over 3 years	130 (45.0)
	Below 3 years	138 (55.0)
GFD adherence ^1^	1	2 (0.8)
	2	31 (12.4)
	3	26 (10.4)
	4	185 (73.7)
	0	7 (2.8)
BMI (kg/m^2^) ^2^	<18.5—underweight	38 (15.3)
	18.5–24.9—normal weight	165 (66.3)
	25–29.9—overweight	46 (18.5)
Place of residence	Village	49 (19.5)
	Town up to 100,000 residents	72 (28.7)
	Cities over 100,000 residents	130 (51.8)
Educational level	Primary and secondary	61 (24.3)
	Postgraduate	57 (22.7)
	University degree	133 (53.0)
Economic status	Very bad	5 (2.0)
	Bad	18 (7.2)
	Average	119 (47.4)
	Good	84 (33.5)
	Very good	25 (10.0)

^1^ GFD (gluten-free diet) adherence: (1) very poor; (2) good, but occasionally eat dishes containing gluten (at home or outside home); (3) very good, but occasionally eat dishes containing gluten (only outside home); (4) excellent; (0) difficult to say; ^2^ *n* = 249.

**Table 2:** Descriptive statistics for the CDQ.

Characteristics	Measure		Emotional (7–49)	Social (7–49)	Worries (5–35)
Score	Mean ± SD		27.2 ± 9.6	36.1 ± 9.7	19.6 ± 6.1
	95% CI		(26.2–28.2)	(34.9–37.3)	(19.6–20.4)
	Median		26.0 ^1^	38.0 ^1^	19.0 ^1^
	Min–max		7.0–49.0	8.0–49.0	6.0–35.0
	25th–75th		22–33	29–45	14–24
Data quality	Floor effect	%	1	0	0
	Ceiling effect	%	1	20	2
Scaling assumptions	Item internal consistency	Item-scale correlation (corrected for overlap)	0.41–0.66	0.38–0.74	0.25–0.48
		Pearson item-scale correlation ≥ 30% ^2^	100%	100%	80%
	Internal consistency reliability	Cronbach’s alpha	0.88	0.88	0.74

^1^ non-parametric distribution (verified using Shapiro-Wilk test—*p* ≤ 0.05); ^2^ according to Everitt & Skrondal [28].

**Table 3:** Emotional subscale data categorized by CD duration, GFD adherence, BMI and sociodemographic characteristics.

Categories		Mean ± SD	Median (min–max)	*p*-Value ^1^
CD duration	Over 3 years	27.1 ± 7.8	27.0 (7.0–47.0)	0.8851
	Below 3 years	27.2 ± 8.3	26.0 (11.0–49.0)	
GFD adherence ^3^	1	26.0 ± 1.4	26.0 (25.0–27.0) ^2^	0.6714
	2	27.1 ± 8.5	24.5 (18.0–48.0) ^2^	
	3	25.2 ± 6.9	23.0 (14.0–42.0)	
	4	27.5 ± 8.2	27.0 (7.0–49.0)	
	0	23.6 ± 7.0	22.0 (15.0–35.0)	
BMI category	Underweight	25.7 ± 9.0	25.0 (7.0–43.0)	0.5127
	Normal weight	27.3 ± 8.1	27.0 (11.0–49.0) ^2^	
	Overweight	27.8 ± 7.2	27.0 (14.0–48.0)	
Place of residence	Village	28.5 ± 6.7	29.0 (13.0–49.0)	0.5299
	Town up to 100,000 residents	26.2 ± 9.1	23.5 (7.0–48.0)	
	Town over 100,000 residents	27.2 ± 7.9	26.0 (11.0–46.0)	
Educational level	Primary and Secondary	25.1 ± 7.6	23.0 (7.0–43.0) ^2^	0.0594
	Postgraduate	25.9 ± 8.0	25.0 (11.0–48.0)	
	University degree	28.6 ± 8.1	28.0 (11.0–49.0)	
Economic status	Very bad and bad	21.5 ± 7.3	21.0 (11.0–42.0) ^2^	0.0015
	Average	26.5 ± 7.8	25.0 (7.0–49.0)	
	Good and very good	29.1 ± 7.8	28.0 (13.0–46.0)	

^1^ multifactor analysis of variance (ANOVA); ^2^ non-parametric distribution (verified using Shapiro-Wilk test—*p* ≤ 0.05); ^3^ GFD (gluten-free diet) adherence: (1) very poor; (2) good, but occasionally eat dishes containing gluten (at home or outside home); (3) very good, but occasionally eat dishes containing gluten (only outside home); (4) excellent; (0) difficult to say.

**Table 4:** Social subscale data categorized by CD duration, GFD adherence, BMI and sociodemographic characteristics.

Categories		Mean ± SD	Median (min–max)	*p*-Value ^1^
CD duration	Over 3 years	35.2 ± 9.6	36.0 (8.0–49.0) ^2^	0.2012
	Below 3 years	36.9 ± 9.7	39.0 (14.0–49.0) ^2^	
GFD adherence ^3^	1	36.5 ± 14.9	36.0 (26.0–47.0) ^2^	0.2526
	2	38.1 ± 9.6	41.0 (14.0–49.0) ^2^	
	3	34.8 ± 10.8	37.0 (14.0–49.0)	
	4	36.2 ± 9.5	38.0 (8.0–49.0)	
	0	30.3 ± 10.4	27.0 (17.0–46.0)	
BMI category	Underweight	35.9 ± 10.8	38.5 (8.0–49.0) ^2^	0.5055
	Normal weight	36.5 ± 9.1	38.0 (14.0–49.0) ^2^	
	Overweight	35.1 ± 11.0	39.0 (11.0–49.0) ^2^	
Place of residence	Village	37.4 ± 9.1	40.5 (17.0–49.0) ^2^	0.2662
	Town up to 100,000 residents	34.2 ± 9.5	33.0 (15.0–49.0) ^2^	
	Town over 100,000 residents	36.7 ± 9.9	39.0 (8.0–49.0) ^2^	
Educational level	Primary and Secondary	33.9 ± 10.6	33.0 (8.0–49.0) ^2^	0.3328
	Postgraduate	35.8 ± 9.4	39.0 (11.0–49.0) ^2^	
	University degree	37.3 ± 9.3	39.0 (14.0–49.0) ^2^	
Economic status	Very bad and bad	28.9 ± 11.2	27.0 (8.0–48.0)	0.0023
	Average	35.7 ± 9.6	37.0 (11.0–49.0) ^2^	
	Good and very good	38.2 ± 8.7	40.0 (16.0–49.0) ^2^	

^1^ multifactor analysis of variance (ANOVA); ^2^ non-parametric distribution (verified using Shapiro-Wilk test—*p* ≤ 0.05); ^3^ GFD (gluten-free diet) adherence: (1) very poor; (2) good, but occasionally eat dishes containing gluten (at home or outside home); (3) very good, but occasionally eat dishes containing gluten (only outside home); (4) excellent; (0) difficult to say.

**Table 5:** Worries subscale data categorized by CD duration, GFD adherence, BMI and sociodemographic characteristics.

Categories		Mean ± SD	Median (min–max)	*p*-Value ^1^
CD duration	Over 3 years	19.1 ± 6.5	18.0 (7.0–35.0) ^2^	0.3352
	Below 3 years	20.0 ± 6.7	20.0 (6.0–35.0) ^2^	
GFD adherence ^3^	1	18.0 ± 2.8	18.0 (16.0–20.0) ^2^	0.2462
	2	21.0 ± 7.1	21.0 (8.0–34.0) ^2^	
	3	19.1 ± 7.7	18.0 (8.0–34.0)	
	4	19.7 ± 6.3	19.0 (7.0–35.0)	
	0	14.3 ± 8.9	13.0 (6.0–33.0) ^2^	
BMI category	Underweight	19.8 ± 7.0	19.0 (8.0–35.0)	0.7485
	Normal weight	19.6 ± 6.4	19.0 (6.0–35.0) ^2^	
	Overweight	19.5 ± 7.2	20.0 (7.0–33.0)	
Place of residence	Village	20.2 ± 7.2	20.0 (8.0–35.0)	0.0816
	Town up to 100,000 residents	18.1 ± 6.0	18.0 (6.0–33.0)	
	Town over 100,000 residents	20.2 ± 6.6	20.0 (7.0–35.0)	
Educational level	Primary and Secondary	18.7 ± 6.9	17.5 (8.0–35.0) ^2^	0.7919
	Postgraduate	19.7 ± 6.4	20.0 (6.0–34.0)	
	University degree	19.9 ± 6.6	19.0 (8.0–35.0) ^2^	
Economic status	Very bad and bad	14.8 ± 5.1	15.0 (6.0–24.0)	0.0015
	Average	19.3 ± 6.7	19.0 (7.0–34.0) ^2^	
	Good and very good	20.9 ± 6.3	21.0 (8.0–35.0)	

^1^ multifactor analysis of variance (ANOVA); ^2^ non-parametric distribution (verified using Shapiro-Wilk test—*p* ≤ 0.05); ^3^ GFD (gluten-free diet) adherence: (1) very poor; (2) good, but occasionally eat dishes containing gluten (at home or outside home); (3) very good, but occasionally eat dishes containing gluten (only outside home); (4) excellent; (0) difficult to say.

**Table 6:** Purchase-related emotions and behaviors data regarding question about mood affecting purchase decisions (*In the past 2 weeks, how many times did your mood affect your purchase decisions associated with GF products?*) categorized by CD duration, GFD adherence, BMI and sociodemographic characteristics.

Categories		Mean ± SD ^4^	Median (Min–Max)	*p*-Value ^1^
CD duration	Over 3 years	4.2 ± 1.8	3.0 (1.0–7.0) ^2^	0.7272
	Below 3 years	4.2 ± 1.9	4.0 (1.0–7.0) ^2^	
GFD adherence ^3^	1	5.0 ± 0.0	5.0 (5.0–5.0) ^2^	0.1981
	2	4.8 ± 1.8	5.5 (2.0–7.0) ^2^	
	3	3.9 ± 1.9	3.0 (1.0–7.0) ^2^	
	4	4.2 ± 1.9	4.0 (1.0–7.0) ^2^	
	0	3.1 ± 1.8	3.0 (2.0–7.0) ^2^	
BMI category	Underweight	4.4 ± 2.0	4.0 (1.0–7.0) ^2^	0.3959
	Normal weight	4.2 ± 1.9	4.0 (1.0–7.0) ^2^	
	Overweight	4.0 ± 1.9	3.0 (1.0–7.0) ^2^	
Place of residence	Village	4.4 ± 1.9	4.0 (1.0–7.0) ^2^	0.2567
	Town up to 100,000 residents	3.9 ± 1.8	3.0 (1.0–7.0) ^2^	
	Town over 100,000 residents	4.3 ± 1.9	4.0 (1.0–7.0) ^2^	
Educational level	Primary and Secondary	4.4 ± 1.8	4.0 (1.0–7.0) ^2^	0.4635
	Postgraduate	3.9 ± 1.7	3.0 (1.0–7.0) ^2^	
	University degree	4.2 ± 2.0	4.0 (1.0–7.0) ^2^	
Economic status	Very bad and bad	3.2 ± 1.7	3.0 (1.0–6.0) ^2^	0.0029
	Average	4.0 ± 1.8	3.0 (1.0–7.0) ^2^	
	Good and very good	4.6 ± 1.9	5.0 (1.0–7.0) ^2^	

^1^ multifactor analysis of variance (ANOVA); ^2^ non-parametric distribution (verified using Shapiro-Wilk test—*p* ≤ 0.05); ^3^ GFD (gluten-free diet) adherence: (1) very poor; (2) good, but occasionally eat dishes containing gluten (at home or outside home); (3) very good, but occasionally eat dishes containing gluten (only outside home); (4) excellent; (0) difficult to say; ^4^ seven point Likert scale, while (1) is attributed to “all of the time” and (7)—to “none of the time”.

**Table 7:** Purchase-related emotions and behaviors data regarding question about purchasing GF products because of mood (*In the past 2 weeks, how many times did you buy GF products to improve your mood?*) categorized by CD duration, GFD adherence, BMI and sociodemographic characteristics.

Categories		Mean ± SD ^4^	Median (min–max)	*p*-Value ^1^
CD duration	Over 3 years	3.9 ± 1.5	4.0 (1.0–7.0) ^2^	0.7936
	Below 3 years	3.9 ± 1.7	3.0 (1.0–7.0) ^2^	
GFD adherence ^3^	1	5.0 ± 0.0	5.0 (5.0–5.0) ^2^	0.2695
	2	3.9 ± 1.6	3.5 (1.0–7.0) ^2^	
	3	3.3 ± 1.4	3.0 (1.0–7.0) ^2^	
	4	4.1 ± 1.7	4.0 (1.0–7.0) ^2^	
	0	3.1 ± 1.4	3.0 (2.0–6.0) ^2^	
BMI category	Underweight	4.1 ± 1.8	4.0 (1.0–7.0) ^2^	0.5132
	Normal weight	4.0 ± 1.6	3.0 (1.0–7.0) ^2^	
	Overweight	3.7 ± 1.6	3.0 (1.0–7.0) ^2^	
Place of residence	Village	4.4 ± 1.7	4.0 (1.0–7.0) ^2^	0.0847
	Town up to 100,000 residents	4.0 ± 1.6	3.0 (1.0–7.0) ^2^	
	Town over 100,000 residents	3.8 ± 1.6	3.0 (1.0–7.0) ^2^	
Educational level	Primary and Secondary	4.1 ± 1.6	3.5 (1.0–7.0) ^2^	0.3801
	Postgraduate	3.6 ± 1.7	3.0 (1.0–7.0) ^2^	
	University degree	4.0 ± 1.6	4.0 (1.0–7.0) ^2^	
Economic status	Very bad and bad	3.2 ± 1.2	3.0 (1.0–7.0) ^2^	0.0605
	Average	3.8 ± 1.6	3.0 (1.0–7.0) ^2^	
	Good and very good	4.2 ± 1.7	4.0 (1.0–7.0) ^2^	

^1^ multifactor analysis of variance (ANOVA); ^2^ non-parametric distribution (verified using Shapiro-Wilk test—*p* ≤ 0.05); ^3^ GFD (gluten-free diet) adherence: (1) very poor; (2) good, but occasionally eat dishes containing gluten (at home or outside home); (3) very good, but occasionally eat dishes containing gluten (only outside home); (4) excellent; (0) difficult to say; ^4^ seven point Likert scale, while (1) is attributed to “all of the time” and (7)—to “none of the time”.
